# New Waste-Based Composite Material for Construction Applications

**DOI:** 10.3390/ma14206079

**Published:** 2021-10-14

**Authors:** Eugen Constantin Ailenei, Savin Dorin Ionesi, Ionut Dulgheriu, Maria Carmen Loghin, Dorina Nicolina Isopescu, Sebastian George Maxineasa, Ioana-Roxana Baciu

**Affiliations:** 1 Faculty of Industrial Design and Business Management, “Gheorghe Asachi” Technical University of Iasi, Blvd. Mangeron, No. 29, 700050 Iasi, Romania; eugen.ailenei@tuiasi.ro (E.C.A.); ionut.dulgheriu@academic.tuiasi.ro (I.D.); 2 Faculty of Civil Engineering and Building Services, “Gheorghe Asachi” Technical University of Iasi-Romania, Blvd. Mangeron, No. 1, 700050 Iasi, Romania; isopescu@tuiasi.ro (D.N.I.); sebastian.maxineasa@tuiasi.ro (S.G.M.)

**Keywords:** waste-based composite, panel, construction applications, textile waste, mechanical properties

## Abstract

The global demand for fiber-based products is continuously increasing. The increased consumption and fast fashion current in the global clothing market generate a significant quantity of pre-and post-production waste that ends up in landfills and incinerators. The present study aims to obtain a new waste-based composite material panel for construction applications with improved mechanical properties that can replace traditional wood-based oriented strand boards (OSB). The new composite material is formed by using textile wastes as a reinforcement structure and a combination of bi-oriented polypropylene films (BOPP) waste, polypropylene non-woven materials (TNT) waste and virgin polypropylene fibers (PP) as a matrix. The mechanical properties of waste-based composite materials are modeled using the Taguchi method based on orthogonal arrays to maximize the composite characteristics’ mechanical properties. Experimental data validated the theoretical results obtained.

## 1. Introduction

Nowadays, the demand for textile products is experiencing exponential growth generated by both demographic indicators and the fashion industry. By increasing consumption and fast-changing fashion trends in the global clothing market as a result of the new fast fashion current, a substantial product life cycle shortening can be remarked. These aspects generate increased amounts of post-consumer waste added to the initial pre-consumption waste, which results during all technological flows of the processing industry of fibers, fabrics, textile garments, interior textiles and technical textiles.

**Figure 1:**
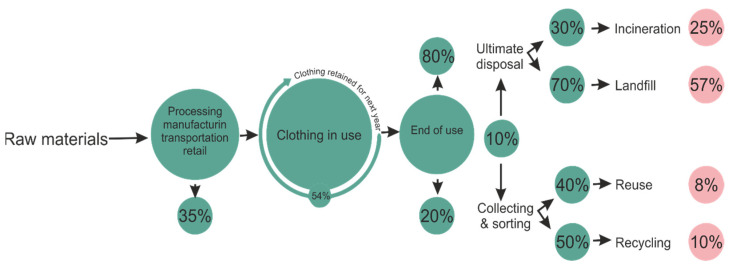
Pre- and post-consumer waste in the clothing industry [1].

According to the analysis made by Boston Consulting Group [1] for the Copenhagen Summit “Pulse of the Fashion Industry 2017” out of a total of 2.1 billion tons of waste produced worldwide annually 4%, representing 92 million tons, are produced by the fashion industry. Over 35% of pre-consumption waste is generated in the primary processing phase of the raw material, of which 9% is in fiber production and 91% in technological operations of product manufacturing (spinning, weaving and manufacturing). This analysis also shows that the processing and recycling of post-consumer textile waste are limited in quantity and technology. In this case, 80% of post-consumer waste ends up in landfills and incinerators, and only 20% is recycled or reused. All these details related to raw materials are presented in graphic form in Figure 1.

According to demographic statistics, in 2030, the world population will be about 8.5 billion persons, and the demand for clothing products is expected to increase from 60 million tons in 2018 by 63% to 102 million tons in 2030 with a significant environmental impact [1].

From the perspective of textile products consumption per person in the industrial countries, the demand in 2010 is at an average of 28 kg/person increasing in 2020 by 10%, at 31 kg/person. Still, demand is growing by 51% in developing countries, from 7.9 to 12 kg/person [2,3,4].

**Figure 2:**
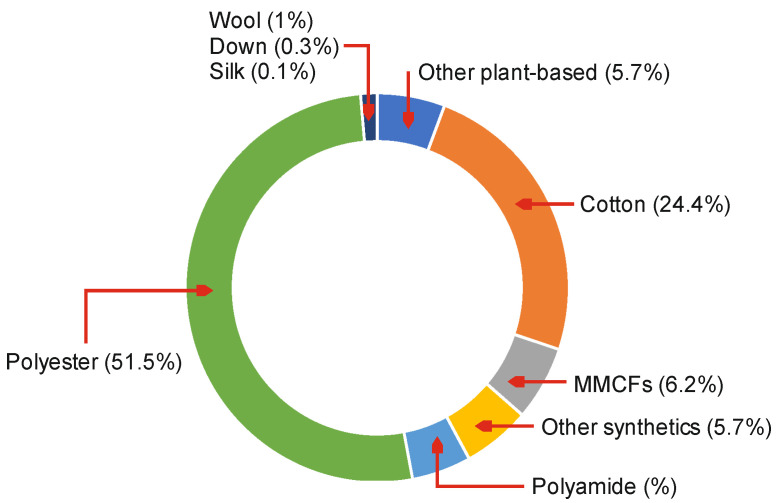
Fiber-type market share [5].

To have a more precise image of the quantity and composition of the waste generated by the textile industry, we will analyze the demand both by type of fibers (natural and chemical) and on the proportion of each type of fibers within this industry. At the level of the 2018 year, the amount of polyester fibers worldwide consumed far exceeded the other types of chemical fibers and those of natural fibers (Figure 2). The share of synthetic fibers is 62.2% and natural fiber 37.7%. From synthetic fibers total consumption, polyester has over 51.5% of total demand. The percentage of fibers used for clothing is a significant share of over 55% [5].

It can be remarked that synthetic fibers have a growing share of the textile industry market. A large part of them is directed to the fashion industry and interior products, generating significant amounts of pre-consumption and post-consumption waste with a very long degradation period. The global trend is to reduce the demand for natural cotton fibers due to the negative impact on the environment. Considering that cotton production consumes an estimated 16% of all insecticides and 7% of all herbicides [1,6], the global trend is to reduce the demand for natural cotton fibers due to the negative impact on the environment. A significant part of this market share has been taken by eco-friendly cotton and polyester fibers with similar characteristics [6,7].

The European Council adopted on 22 May 2018 the waste management package of laws: Directive 2018/851 of the European Parliament and of the Council of 30 May 2018 amending Directive 2008/98/EC on waste, which lays down new rules on waste management and new recycling targets. According to these directives, the member states shall establish, by 1 January 2025, the separate collection of textiles and hazardous waste from households [8].

The main methods used for textile materials recycling, according to Ellen MacArthur Foundation [6,7], are classified as follows:

- Mechanical—textile waste is transformed into new products without changing the basic chemical structure by cutting, shredding and defibrating the waste to the level of fibers and transforming them into yarns and fabrics. It is closed-loop recycling with the addressability of all types of fibers (vegetable, animal and oil-based). The new products obtained are new yarns and non-woven materials. The qualities of the fibers obtained are generally inferior to the virgin fibers used in the initial products.
- Chemical—textile waste is decomposed into monomers, oligomers or basic chemicals. This form of recycling produces high-value products because they are identical to the original constituents used in obtaining the products subject to recycling. In addition, it addresses vegetable and oil-based fibers.
- Thermal—often refers to transforming PET flakes, pellets or chips into fibers by extruding the melt. It is often confused with energy recovery by burning textile waste.

The recycling process often consists of a mixture of mechanical, chemical and thermal processes in most cases. Examples are chemical recycling methods, in which, before dissolution or depolymerization, in the case of cellulosic fibers or monomers/oligomers, they are mechanically processed. This is similar to thermal recycling because the production of flakes, pellets and chips from PET waste is carried out by mechanical means. To remove paint pigments, additives and other impurities, chemical treatments are performed before mechanical recycling [9]. The recycling process can be in a closed or open cycle, according to the resulted product. For example, suppose the textile waste is converted into the same type as the initial one. In that case, it can be affirmed that it is a closed recycling cycle by recovering both components [10,11] or just the polyester polymer [12,13].

On the other hand, suppose the textile waste is converted into a new product with a different destination. In that case, the recycling cycle is open, used to make thermal insulation materials and sound-absorbing material for construction applications [14,15,16] or composite materials with thermoplastic matrix [17,18,19,20]. In addition, they are found with a reinforcing role in different applications for the realization of composite materials used in various sectors [19,20,21,22,23].

Considering the low recycling rate of waste resulting from pre-and post-consumption of textile products, the researchers are constantly looking for new environmentally friendly recycling technologies [18,24,25] and new applications for waste-based textile products [26,27,28]. The general direction is to upcycle, to obtain new products with increased added value and functionalities, but in general practice, the recycling of textile waste is downcycled [29,30].

The main objective of the completed research was to assess the possibility of reducing the negative impact of the textile and building industries on the natural environment by manufacturing a panel made of textile wastes that can be used for replacing the oriented strand boards (OSB) in different civil engineering applications. The 8 mm OSB is the most suitable construction product for comparison due to the fact that it is highly popular for building interior non-structural walls in houses made by using the light timer framing system. Therefore, the authors compared the analyzed textile waste product only with the 8 mm OSB, and thus, the composite product has the same thickness.

## 2. Materials and Methods

To complete this research have been used unsorted and underrated textile pre- and post-consumption wastes as reinforcement structure and bi-oriented polypropylene films (BOPP) waste, polypropylene non-woven materials (TNT) waste and virgin polypropylene fibers (PP) as the matrix.

The experimental work focused on producing textile-reinforced composite materials entirely made from production and technological waste for both reinforcement structures and matrices using thermoforming technology. This process is the adequate technology of the product targeted in work due to its advantages: short technological flow; low costs; no operations needed for sorting the textile wastes; elimination of the process of defibration of the textile wastes, which is an energy-intensive operation.

The investigation work has been carried out using the Taguchi Design of Experiment (DOE) method based on orthogonal arrays that use performance indicators, such as the signal-noise (S/N) ratio, that simultaneously take into consideration the desired response value (signal) and the variability thereof (noise) [31,32,33]. The DOE method aims to minimize the variability of the parameters reported to the noise factors and maximize the variability reported to the signal factors. The main steps that were followed to complete the experiment are:

- definition of system objectives—can be represented either by parameter optimization either by reaching a minimum or maximum value. The deviation from optimum performance is used to define the quality loss function;
- determination of parameters that influence the system and the specific levels of each;
- definition of the orthogonal array used in experimenting, according to the number of parameters and their specific levels;
- implementation of experiment and collect the experimental data;
- statistically analysis and interpretation of obtained raw data;
- results validation.

Taking into consideration the main objective of the research, the S/N ratio has been determined using the more significant, the better relation:

(1) SN=−10log(∑(1Y2)/n)

where *Y* = responses for the given factor level combination and *n* = number of responses in the factor level combination.

The main signal parameters taken into consideration are represented by matrix type, temperature, time, pressing force and matrix proportion. The specific levels of each are presented in Table 1.

After analyzing orthogonal array models, the signal parameters and specific levels of each have been deemed adequate for the L16 orthogonal array, presented in Table 2 in coded (C) and uncoded (UNC) values.

**Figure 3:**
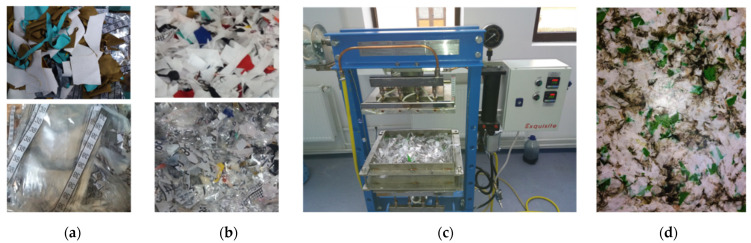
The stages of the technological thermoforming process: (**a**) pre-and post-production waste; (**b**) cutting; (**c**) mixing, thermoforming and cooling; (**d**) waste-based composite panel.

**Figure 4:**
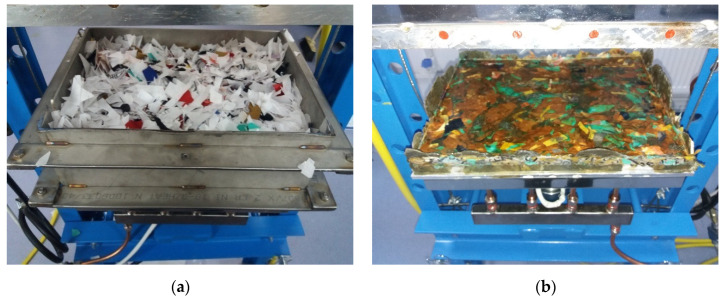
The waste-based composite materials panels: (**a**) pre-and (**b**) post-the technological thermoforming process.

The waste-based composite materials panels were made using thermoforming technology, as illustrated in Figure 3 and Figure 4. The used thermoforming press uses two plates that can be electrically heated up to 250 °C and water-cooling systems. The main parameters that can be modified are represented by temperature (0–250 °C) and pressing force (0–196 N/cm^2^).

The mechanical properties of the obtained waste-based composite materials were evaluated from a tensile and flexural point of view using the LBG TC10 testing machine equipped with a 10kN load cell, taking into consideration the specifications of SR EN 300:2006 [34] and SR EN ISO 527-4:2000 standards [35]. For each sample, a series of five tests were performed according to the standards mentioned above. In addition, the SR EN 300:2006 standard has been used to compare with 8 mm OSB boards.

The reduction by 20% of the distance between supports has been decided because the samples subjected to flexure did not break. To record the maximum bending force in 60 ± 30 s, the speed test was set at 20 mm/min.

According to SR EN 310-1996, the bending strength of composite samples is calculated as a ratio between the bending moment (at the maximum load *F_max_*) to the moment of its full cross-section:

(2) $$f_{m}=\frac{3F_{max}}{2bt^{2}}$$

where:

*f_m_* = bending strength [N/mm^2^];
*F_max_* = maximum load [N];
*l*_1_ = distance between the supports [mm];
*b* = width of the test sample [mm];
*t* = thickness of the test sample [mm].

According to SR EN 310-1996, Young’s modulus has been determined for each sample using the following relation:

(3) $$E_{m}=\frac{l_{1}^{3}x\left(F_{2}-F_{1}\right)}{4br^{2}x\left(a_{2}-a_{1}\right)}$$

where:

*l*_1_ = distance between the supports [mm]
*b* = width of the test sample [mm];
*t* = thickness of the test sample [mm];
*F*_2_ − *F*_1_ = increasing the force, in newtons, on the straight portion of the load-deformation curve. *F*_1_ is 10% of the breaking load, and *F*_2_ is 40% of the breaking load.
*a*_2_ − *a*_1_ = the increase of the arrow at the middle of the sample (corresponding to *F*_2_ − *F*_1_)

**Figure 5:**
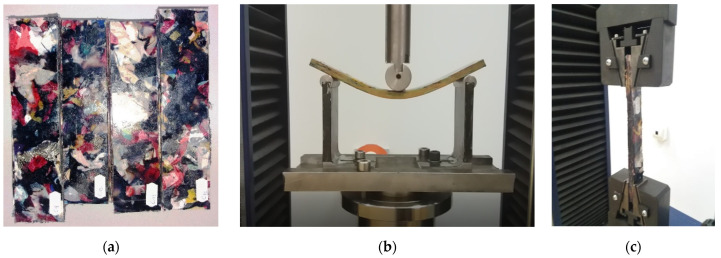
Mechanical properties testing (C9, C10, C11, C12): (**a**) tested samples; (**b**) compression testing; (**c**) stretch testing.

According to SR EN ISO 527-4:2000, the tensile strength has been automatically determined using testing samples type 2, as illustrated in Figure 5.

## 3. Results

The samples were produced according to the proposed experimental matrix, and the density, bending, Young’s modulus and tensile strength were determined. The definition of the signal and noise factors is followed by statistical analysis. The results are presented in Table 3.

The response resulting from Taguchi analysis revealed the significance of the signal factors reported to the S/N ratio and the mean. The obtained regression results are presented in Table 4, for the S/N ratio and in Table 5, for means. Combining the obtained results from S/N ratio analysis and means analysis results in the classification of influence level of the factors over the proposed model, presented in Table 6.

By analyzing the regression results for S/N ratio and means, illustrated in Table 4 and Table 5 and the response resulting from Taguchi analysis, presented in Table 6, the significance of the signal factors reported to S/N ratio, and the means is:

- Maximum influence—factor E (matrix proportion);
- High influence—factor A (matrix type);
- High influence—factor C (time);
- Low influence—factor D (pressing force);
- Low influence—factor B (temperature).

**Figure 6:**
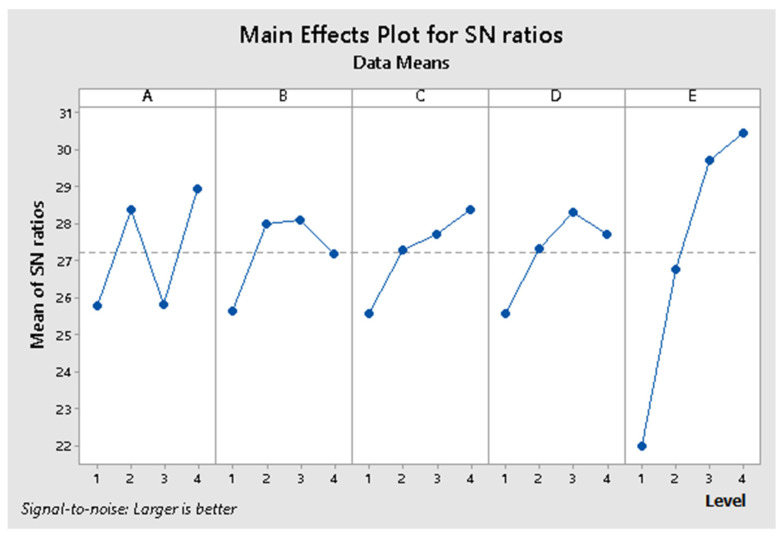
The values obtained for the main effects plot for S/N ratio.

**Figure 7:**
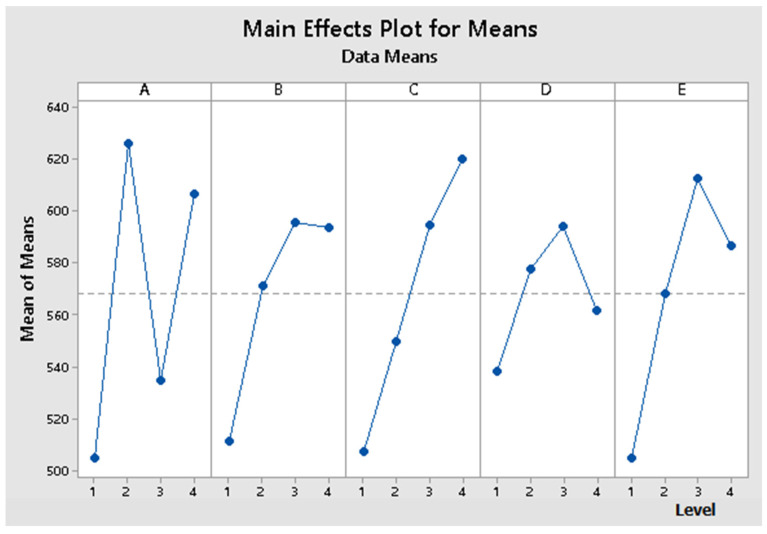
The values obtained for the main effects plot for means.

**Figure 8:**
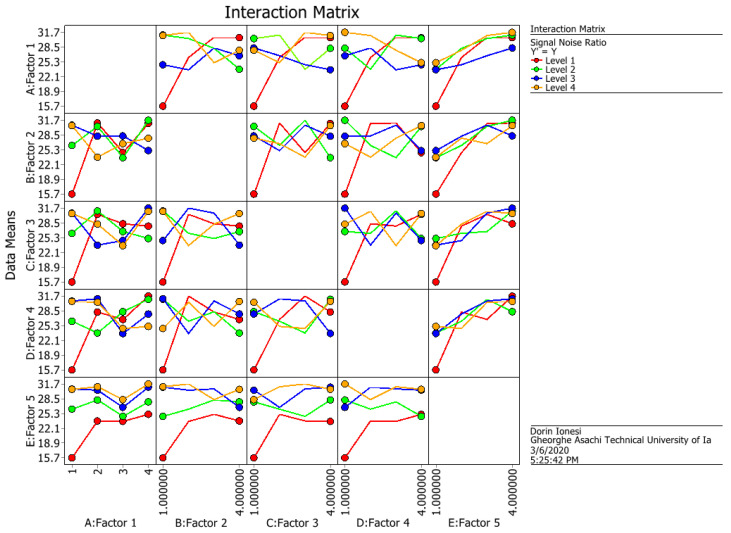
The interactions between factors for S/N ratio.

**Figure 9:**
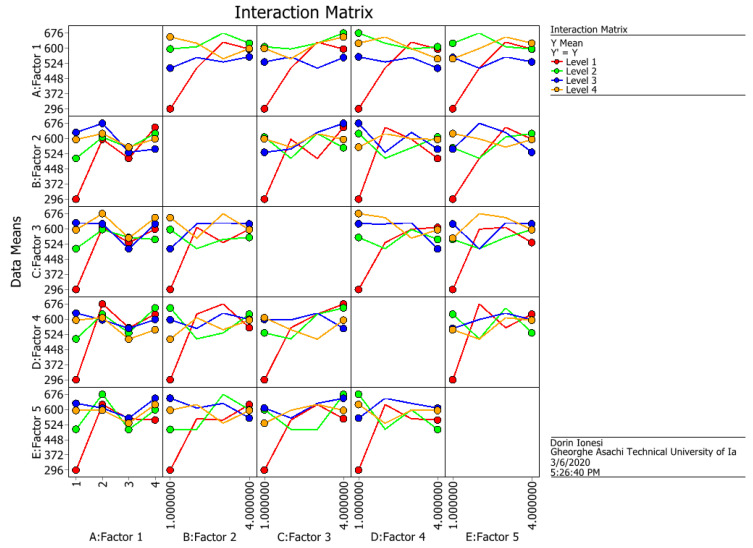
The interactions between factors for means.

The values obtained for the S/N ratio and means from Table 3 are graphically represented in Figure 6 and Figure 7, and the interactions between factors are graphically represented in Figure 8 and Figure 9.

The values obtained for the S/N ratio and Table 3 are graphically represented in Figure 6 and Figure 7. The interaction matrix for the S/N ratio and means are illustrated in Figure 8 and Figure 9. The S/N ratio has been calculated using the formula defined for larger, the better case.

By analyzing the results obtained for the S/N ratio, the optimal combination of signal parameters is A4 B2 C4 D3 E4, representing a composite material that uses the BOPP + TNT as matrix, a temperature of 190 °C, thermic treatment time of 25 min, 108 N/cm^2^ and 50% Matrix proportion. By analyzing the results obtained for means, the optimal combination of signal parameters is A4 B3 C4 D3 E3, representing a composite material that uses the BOPP + TNT as the matrix, a temperature of 180 °C, thermic treatment time of 25 min, pressing force 108 N/cm^2^ and 40% Matrix proportion.

From the S/N ratio point of view, it can be remarked that factor E has a low variation on the 3rd and 4th levels. Still, according to means analysis made across the same levels, the 3rd one has a high influence over the properties of the designed composite material. Therefore, by combing the obtained results for S/N ratio and means analysis, the optimum signal factors combination is A4 B2 C4 D3 E3, representing a composite material that uses the BOPP + TNT as a matrix, a temperature of 190 °C, thermic treatment time of 25 min, pressing force 108 N/cm^2^ and 40% Matrix proportion.

**Figure 10:**
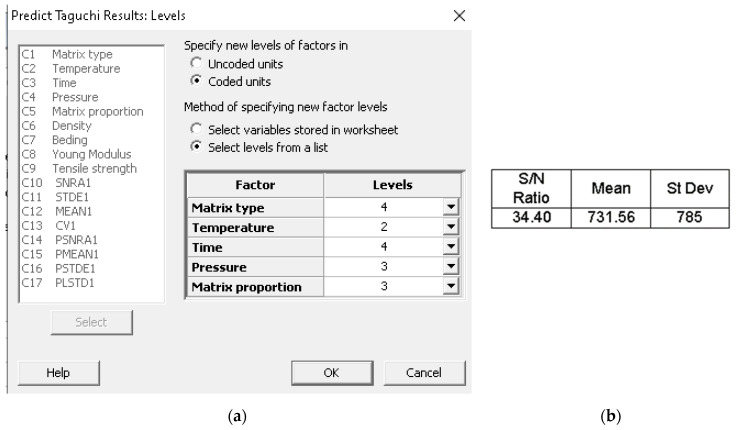
Definition of the levels of the parameters for (**a**) result prediction; (**b**) predicted values.

As shown in Figure 10, after applying the prediction method for the optimal model, the S/N ratio is maximal reported to the S/N ratio obtained from statistical processing of experimental data according to the Taguchi experiment matrix.

This method can be used for predicting results characterized by different combinations of the considered signal parameters.

## 4. Model Validation

For the determination of the accuracy of Taguchi analysis, model validation has been made. In this case, model validation was made by performing several tests, taking into account the optimum levels previously determined of the defined signal parameters (Table 7).

The mechanical properties of the obtained optimum model of the waste-based composite material were evaluated from the tensile and flexural point, considering the specifications of SR EN 300:2006 and SR EN ISO 527-4:2000 standards.

It can be remarked that the obtained results are maximum from a tensile strength point of view, and lower with 5% from Young Modulus point of view (reported to A7 sample) and with 1% from Bending point of view (reported to A13 sample), but with a density lower with 13% reported to the maximum value (A3 sample).

Considering that the waste-based composite material has been designed as an alternative for 8 mm OSB boards, the obtained mechanical properties were compared to the mechanical properties of the 8 mm OSB boards (Table 8). Therefore, the OSB board’s mechanical properties, presented in Table 8, were determined according to SR EN 300:2006 and SR EN ISO 527-4:2000 standard specifications.

The reinforcement structure into the designed waste-based composite material was randomly arranged. Due to this fact, the mechanical properties are isotropic.

By comparing the mechanical properties of the designed waste-based composite material to the ones of the 8 mm OSB boards, it can be remarked that:

- the tensile strength of waste-based composite material is higher with 1030.97% on the transversal direction and 410.21% on longitudinal direction;
- the bending resistance of waste-based composite material is higher with 351.92% on the transversal direction and 150% on longitudinal direction;
- the rigidity of waste-based composite material is lower with 8% on the transversal direction and with 60% on longitudinal direction;
- the density of waste-based composite is higher at 173%.

## 5. Conclusions

The present paper aims at assessing the opportunity of improving the overall environmental performances of the construction and textile industries by using various textile wastes for manufacturing a panel that can be used as an OSB board replacement, with a thickness of 8 mm, for assembling the interior non-structural walls in light timber frame building. The novelty of this study resides in the combination of pre-and post-production textile wastes used as reinforcement structure and bi-oriented polypropylene films waste and polypropylene non-woven materials waste used as matrix.

The obtained results reveal that developing composite material panels for construction applications made from pre-and post-production textile material wastes to replace traditional wood-based oriented strand boards is an efficient solution. In addition, the mechanical properties of the new material are significantly higher than those of traditional 8 mm OSB boards.

The use of virgin polypropylene fibers as a matrix brings no advantage compared to bi-oriented polypropylene films waste and polypropylene non-woven materials waste. Therefore, the new waste-based composite material was validated based on experimental results obtained by testing the resulting optimal model in similar conditions and comparing the obtained results with the initial model and 8mm OSB board mechanical characteristics.

## Figures and Tables

**Table 1:** Signal parameters selection.

Level	Matrix Type	Temperature °C	Time [min]	Pressing Force [N/cm^2^]	Matrix Proportion [%]
**1**	BOPP	180	10	68	20
**2**	TNT	190	15	88	30
**3**	PP	200	20	108	40
**4**	BOPP + TNT	210	25	128	50
**Symbol**	A	B	C	D	E

**Table 2:** Orthogonal array selection.

Experiment Annotation	Matrix Type (A)		Temperature [°C] (B)		Time [min] (C)		Pressing Force [N/cm^2^] (D)		Matrix Proportion [%] (E)	
	C	UNC	C	UNC	C	UNC	C	UNC	C	UNC
**A1**	1	BOPP	1	180	1	10	1	68	1	20
**A2**	1	BOPP	2	190	2	15	2	88	2	30
**A3**	1	BOPP	3	200	3	20	3	108	3	40
**A4**	1	BOPP	4	210	4	25	4	128	4	50
**B5**	2	TNT	1	180	2	15	3	108	4	50
**B6**	2	TNT	2	190	1	10	4	128	3	40
**B7**	2	TNT	3	200	4	25	1	68	2	30
**B8**	2	TNT	4	210	3	20	2	88	1	20
**C9**	3	PP	1	180	3	20	4	128	2	30
**C10**	3	PP	2	190	4	25	3	108	1	20
**C11**	3	PP	3	200	1	10	2	88	4	50
**C12**	3	PP	4	210	2	15	1	68	3	40
**D13**	4	BOPP + TNT	1	180	4	25	2	88	3	40
**D14**	4	BOPP + TNT	2	190	3	20	1	68	4	50
**D15**	4	BOPP + TNT	3	200	2	15	4	128	1	20
**D16**	4	BOPP + TNT	4	210	1	10	3	108	2	30

**Table 3:** Experimental design using L16 array and experimental results.

No.	A	B	C	D	E	Density [kg/m^3^]	Bending [N/mm^2^]	Young Modulus [N/mm^2^]	Tensile Strength [N/mm^2^]	SNRA	STDE	MEAN	CV
A1	1	1	1	1	1	936	4.7	239.0	3.95	15.66	440.89	295.95	1.49
A2	1	2	2	2	2	1104	19.8	865.9	12.04	26.27	567.89	500.47	1.13
A3	1	3	3	3	3	1283	33.0	1183.7	19.98	30.68	697.89	629.86	1.11
A4	1	4	4	4	4	1075	31.0	1253.1	20.19	30.59	661.27	594.78	1.11
B5	2	1	2	3	4	1065	32.4	1264.2	21.74	31.15	661.68	595.73	1.11
B6	2	2	1	4	3	1118	32.5	1256.9	19.25	30.40	673.04	606.65	1.11
B7	2	3	4	1	2	1220	31.4	1437.4	14.22	28.27	759.10	675.64	1.12
B8	2	4	3	2	1	1136	18.1	1333.8	8.45	23.70	710.05	624.21	1.14
C9	3	1	3	4	2	1024	14.7	948.6	10.56	24.69	562.93	499.42	1.13
C10	3	2	4	3	1	1051	15.6	1137.0	8.65	23.59	625.61	553.03	1.13
C11	3	3	1	2	4	1115	25.9	966.4	15.14	28.34	592.12	530.60	1.12
C12	3	4	2	1	3	1111	22.4	1079.7	12.28	26.66	622.47	556.28	1.12
D13	4	1	4	2	3	1216	35.2	1350.7	20.73	31.06	726.99	655.74	1.11
D14	4	2	3	1	4	1042	35.1	1397.5	23.19	31.75	702.65	624.53	1.13
D15	4	3	2	4	1	1106	21.2	1048.9	9.98	25.14	613.59	546.59	1.12
D16	4	4	1	3	2	1092	27.7	1257.3	13.78	27.85	669.76	597.83	1.12

**Table 4:** Regression Information—S/N ratio.

Term	Coefficient	Standard Error	Low Confidence	High Confidence	T [Value]	*p* [Value]
Intercept	27.24	0.52	26.27	28.20	52.59	0.00
A [1]	−1.44	0.90	−3.11	0.23	−1.60	0.15
A [2]	1.14	0.90	−0.52	2.81	1.27	0.24
A [3]	−1.42	0.90	−3.08	0.25	−1.58	0.15
B: Factor 2	0.72	0.69	−0.58	2.01	1.03	0.33
C: Factor 3	1.33	0.69	0.03	2.62	1.91	0.09
D: Factor 4	1.10	0.69	−0.19	2.39	1.58	0.15
E: Factor 5	4.23	0.69	2.94	5.53	6.09	0.00

**Table 5:** Regression Information—Means.

Term	Coefficient	Standard Error	Low Confidence	High Confidence	T [Value]	*p* [Value]
Intercept	567.96	11.84	545.93	589.98	47.95	0.00
A [1]	−62.69	20.51	−100.84	−24.54	−3.06	0.02
A [2]	57.60	20.51	19.45	95.75	2.81	0.02
A [3]	−33.12	20.51	−71.27	5.02	−1.61	0.15
B: Factor 2	40.38	15.89	10.83	69.93	2.54	0.03
C: Factor 3	57.13	15.89	27.58	86.68	3.60	0.01
D: Factor 4	13.15	15.89	−16.40	42.70	0.83	0.43
E: Factor 5	43.23	15.89	13.68	72.78	2.72	0.03

**Table 6:** Classification of influence level.

Level	Matrix Type (A)	Temperature [°C] (B)	Time [min] €	Pressing Force [N/cm^2^] (D)	Matrix Proportion [%] €
**1**	25.8	25.64	25.56	25.58	22.02
**2**	28.38	28	27.3	27.34	26.77
**3**	25.82	28.11	27.7	28.32	29.7
**4**	28.95	27.2	28.38	27.7	30.46
**Delta**	3.15	2.74	2.81	2.73	8.43
**Rank**	2	5	3	4	1

**Table 7:** Mechanical properties of the obtained optimum model of the waste based composite material.

A	B	C	D	E	Density [kg/m^3^]	Bending [N/mm^2^]	Young Modulus [N/mm^2^]	Tensile Strength [N/mm^2^]
4	2	4	3	3	1112	34.8	1385.6	23.3

**Table 8:** Mechanical properties of the 8 mm OSB boards.

Testing Direction	Density [kg/m^3^]	Bending [N/mm^2^]	Young Modulus [N/mm^2^]	Tensile Strength [N/mm^2^]
Transversal	643	9.9	1499	2.26
Longitudinal	643	23.2	3427.7	5.68

## Data Availability

Not applicable.
